# Annotations

**Published:** 1893-09-16

**Authors:** 


### Annotations.

**Reckless Cycling.**

Last week the Editor of this journal, who is on a holiday, was knocked down and rendered unconscious by the reckless riding of a youthful cyclist. The young man was out on his machine in the dark, without a light, and going at full speed. A friend who was with him warned Mr. Burdett, who moved to the left side, but the cyclist bore full down upon him, with the result that he was knocked down, and rendered unconscious; and found himself unable to walk, and almost to move. An examination of the injuries by Mr. Thomas Bryant revealed severe sprains of muscle and tendon, necessitating confinement to the house o its precincts for some time to come. The offending cyclist, who expressed neither regret nor apology, was captured, and escaped all further consequences of his misdeeds by the payment of a fine of ten shillings, inflicted by the Hampshire magistrates. Circumstances of this kind are of exceedingly common occurrence in free and merry England; and, from the point of view of the youthful cyclist, they may be regarded as a piquant kind of sauce to the day's ride. When a man of middle age and responsible position can be maimed and placed in danger of his life for ten shillings, it cannot be considered a costly species of cycling fun. We may be permitted, however, to point out that there is another side to the question. If the magistrate who imposed a fine of ten shillings had himself been rendered unconscious, and disabled for a month, he would probably have insisted upon a somewhat sterner administration of the law. There are two things here to be demanded in the interests of the public safety; first, that magistrates shall compel respect for the law by imposing adequate fines when it is broken. The young cyclist who knocked Mr. Burdett down was not only riding without a light in a dark country lane, but he was on the wrong side of the road, and he did not take the trouble to ring his bell. Surely here was a case of gross contempt for the public safety, which could only have been adequately punished by the severest penalty the magistrate had it in his power to inflict. Secondly, we would appeal to responsible cyclists, and to the heads of cycling clubs, to protect themselves and their class against the rising indignation of an outraged public. In the long run it is the public who make the laws, and if cyclists will not pay some regard to the safety and convenience of others who are entitled to use the public roads as well as themselves, they will find that much of their freedom, and many of their very abundant privileges, will be taken from them. This is a world of give and take, and neither mad cyclists nor mad dogs can be permitted to play their pranks, unchecked, to the end of time.

**How to "Dish" the Food Adulterator.**

At the provincial meeting of the Incorporated Society of Medical Officers of Health, held recently at Newcastle-on-Tyne, Mr. Alfred Hill, M.D., F.I.C., Medical Officer of Health for Birmingham, delivered an address on "The Influence of Food Adulteration on Health." Among the articles mentioned by Dr. Hill as constituents of our daily food were alum, potash, copper, boric acid, salicylic acid, sulphurous acid, benzoic acid, and many others. On the general question of these adulterations, Dr. Hill remarks: "Supposing that the dose of alum in bread, potash in cocoa, copper in vegetables, boric acid, and borax, salicylic acid, sulphurous acid, and many other strange chemical compounds in almost every kind of food and drink, are not directly and palpably poisonous in their effects, still it cannot be imagined that their repeated and more or less constant ingestion is without danger and injury. . . . The habitual administration of these drugs is probably the cause of many of the obscure ailments which are a great trouble to the victim and an inscrutable mystery to the physician. But so long as scientific men of eminence can be found to support with their sworn evidence such malpractices there is little hope of improvement." Readers may be reminded that Dr. Hill was addressing a scientific gathering, and that there is, therefore, every reason to believe that he spoke with reason and sobriety. We do not desire to indulge in a melancholy wail over these things. Our advice is addressed to the community at large, and it is this: "Let us return to the ways of our forefathers. Many of us can bake our bread at home; we can refuse to drink doubtful cocoa; we can eschew wine of all sorts; we can avoid pickles; we can exclude preserved meats, canned lobsters, fish, *et hoc genus omne* from our tables; we can ostracise bought jams; and generally we can, by a little self-denial and the prospect of great improvement in our health, make it hot for the unscrupulous adulterator by refusing to buy his wares. If this advice be acted upon on a large scale the adulterator will find his adulterating life not worth living and will be compelled to seriously consider the question of turning an honest man.

**Wanted, a Medical Defoe.**

"We sometimes wish," says a writer in the *Spectator*, that "Defoe were alive again. . . . He would show us a short and easy way with blacklegs; he would prove that the true reason for low wages is redundant population, and he would preach on the necessity of raising the community to a higher level by killing out every fifth man." Here are some principles which might easily be applied to the medical profession, and which, if thoroughly applied, would have the inestimable result of "raising the whole medical community." There is no doubt whatever that the profession has its full share, and perhaps more than its full share, of "blacklegs," that is, of legally qualified persons who undersell their neighbours so shamefully that they reduce both themselves and many of their professional brethren to a level of poverty and hard work which is little better than sordid. Defoe's imaginary suggestion of killing out every fifth man among doctors is admirable. But how is it to be done? It is to be done by looking after the examining boards. There is the secret. Now there are several sorts of examining boards which help to crowd the profession with unsuitable and unworthy persons; and chief among these are those boards which conduct preliminary examinations in arts. We do not hesitate to say that one medical student in every five, in other words, "every fifth man," is entirely unsuitable by early education for the ranks of medical studentship. It is here, in fact, where every fifth man should be professionally killed. Medical and surgical examiners are very loth to permanently pluck a man after he has spent three, four, or five years at medical study, and incurred hundreds of pounds of expense. Being men and fathers they have sympathy with other men and fathers. But the parents of boys have incurred very little special expense in having their sons prepared for the preliminary examinations in arts. These examinations should be raised to a very much higher level; should, in fact, correspond to a pass degree in arts at the Universities. If this change were effected the standard of medical education and of the medical profession would be raised throughout; medical life would be worth living; and the public would be greatly advantaged by having doctors of higher competence and worthier character as the medical guardians of health and home. We commend these suggestions to cultured medical students and young practitioners.

